# Breaking silos, building bridges: leveraging Global Collaborative Evidence Networks for global health impact

**DOI:** 10.3389/fpubh.2026.1837626

**Published:** 2026-07-08

**Authors:** Bianca Pilla, Zoe Jordan, Dru Riddle, Miloslav Klugar, Mbah Patrick Okwen, Heidi Parisod, Kavita Kachroo, Susan Weeks

**Affiliations:** 1 JBI, School of Public Health, University of Adelaide, Adelaide, SA, Australia; 2 JBI TCU Center for Translational Research, Cochrane TCU Center, Cochrane US Network, Texas Christian University, Fort Worth, TX, United States; 3 Cochrane Czech Republic, JBI Czech Republic, Czech GRADE Network, Institute of Health Information and Statistics of the Czech Republic, Prague, Czechia; 4 JBI Center of Evidence-Based Education and Arts Therapies, Palacký University Olomouc, Olomouc, Czechia; 5 JBI Effective Basic Services (eBASE) Africa, Bamenda, Cameroon; 6 JBI Finnish Centre for Evidence-Based Health Care, Helsinki, Finland; 7 Nursing Research Foundation, Helsinki, Finland; 8 JBI Kalam Institute of Health Technology, Visakhapatnam, Andhra Pradesh, India

**Keywords:** brokerage, collaboration, evidence-based healthcare, Global Collaborative Evidence Networks, multiplexity

## Abstract

**Introduction:**

Global Collaborative Evidence Networks (GCENs), such as JBI, Cochrane, Campbell, the Guidelines International Network, and the Africa Evidence Network, bring together diverse actors to produce, synthesise, and disseminate evidence to inform policy and practise. Each is a distinct network in its own right, yet how these networks collaborate across networks remains underexplored, and persistent equity disparities in governance, agenda-setting, and capacity continue to constrain their collective impact. This study explores the challenges, facilitators, and benefits of collaboration across GCENs, drawing on participants recruited through the JBI Collaboration (JBIC), many of whom held active membership across multiple GCENs.

**Methods:**

A two-phase mixed-methods design was used. Phase 1 comprised an online survey of 213 JBI Collaboration Entity Directors, Convenors, and Deputies, analysed using descriptive statistics, cross-tabulation, and Pearson correlation coefficients. Phase 2 comprised a moderated director panel (*N* = 5) representing five countries across multiple World Bank income classifications, analysed using Braun and Clarke’s reflexive thematic analysis.

**Results:**

Four themes emerged: navigating barriers, fostering synergy, amplifying impact through multiplex ties, and harmonising efforts. Collaboration is impeded by fragmented governance, resource constraints, and methodological duplication, yet is meaningfully enhanced by boundary-spanning brokers, face-to-face relationship-building, and shared strategic goals.

**Discussion:**

These exploratory findings offer a preliminary evidence base to inform a governance agenda for Global Collaborative Evidence Networks, in which formalised brokerage structures, strategic alignment, and equity-centred design may support the development of a more cohesive global evidence ecosystem.

## 1 Introduction

Central to the global evidence-based healthcare agenda is understanding that progress is reliant upon individuals and organisations working together within a functioning evidence ecosystem (1), and also understanding that the challenges in promoting and supporting this endeavour are diverse, complex, varied between countries and cultures, and require strategies specific to a local context.

This is where networks play a pivotal role. Global Collaborative Evidence Networks (GCENs) bring together diverse actors from within and across evidence ecosystems to share information and expertise and to strengthen capacity for knowledge generation, transfer, and use (1). As a form of collaborative network, a GCEN comprises largely autonomous, geographically distributed, and heterogeneous entities (both organisations and individuals) that collaborate around a unifying purpose, sustained commitment, and a willingness to share information, resources, and accountabilities (2). What distinguishes a GCEN from a single project team or a one-off review collaboration is the combination of a formalised, transnational, and enduring structure with a shared mission to produce, synthesise, and mobilise evidence to inform policy and practise. We use the term as an analytical umbrella for this class of networks; the organisations grouped under it do not necessarily describe themselves as “GCENs.” GCENs like JBI, Cochrane, Campbell, GIN, GRADE Working Group, Africa Evidence Network, WHO Collaboration and many others, whilst differing in form and function, were all established with a common goal: to produce, synthesise and disseminate evidence (either themselves or in partnership with others) to inform policy and practise to improve lives (1). The last 30 years have seen each of these networks (independently and collectively) profoundly impact the global evidence-based agenda. As we pass the midway point for the 2030 United Nations Sustainable Development Goals and a global commitment to ‘leave no one behind’, the interconnected global crises of humanitarian disasters, climate change and health pandemics alongside the changing patterns in globalisation and global interconnectedness all highlight the urgency to strengthen local and global evidence ecosystems to address these societal challenges (3, 4). This includes strengthening ties *across* GCENs to reduce duplication of effort. In the wake of COVID-19, a report by the Global Commission on Evidence to Address Societal Challenges challenged these GCENs to better coordinate in delivering high-quality evidence to support these objectives (5).

Equity issues also become more prominent in a crisis. Whilst CGENs have played a vital role in advancing equity agendas, significant disparities persist. For example, critics’ COVID-19 map of global evidence infrastructures highlights persistent inequalities in governance, agenda setting, evidence access, and authorship recommendations (6, 7). Oliver et al. found that international systematic review networks remain dominated by high-income countries with inadequate methods and processes for supporting responses to LMIC systems and policy questions or appropriate mechanisms for building capacity (8, 9). These structural dynamics extend beyond capacity to governance itself: Northern institutions have been shown to maintain disproportionate control over research agenda-setting, funding distribution, and authorship outputs in global health networks, leaving Global South partners in positions of dependency rather than mutual collaboration (10, 11). Whilst moderately positive progress has been demonstrated in the representation of low- and middle-income country researchers as first authors of research outputs (12), there is still significant room for improvement, and scholars have called for a shift towards co-production, participatory governance, and equitable knowledge and capacity sharing (13, 14). Scholars have further argued that this requires not only reformed practises but also a structural shift in power, including decolonising global health research to ensure that governance, funding, and knowledge production are no longer disproportionately shaped by Western institutions (15). For GCENs to realise their full potential, equity *must* be positioned not only as an outcome but as a guiding principle embedded in their design, leadership, and operations (5, 16).

Beyond evidence-based healthcare, interest in networks across multiple disciplines and sectors, as collaborating, professionalised structures, continues to grow. A large and growing body of empirical research demonstrates that social and professional relationships, as well as the networks they constitute, are influential in explaining the processes of knowledge creation, diffusion, uptake, and use (17). For example, at the interpersonal level, psychologists and organisational behaviour scholars have studied the influence of social networks on the relational quality of knowledge sharing between individuals (18, 19). At the organisational level, management scholars examine how networks within and beyond teams influence how they exchange, combine, and create knowledge (20). At the inter-organisational level, researchers have examined how the characteristics of strategic alliances within networks affect inter-organisational knowledge transfer, absorptive capacity, and innovation (21).

Formal networks can transcend sectors and “have no boundaries [to] cut across the organisation” (22) (p.33). This organisational form is significant considering the functional and multi-regional organisations predominant today. Specifically, GCENs bring together diverse actors from across evidence ecosystems, facilitating information sharing and enabling growth in shared capacities (23, 24), providing opportunities to exploit existing knowledge, gaining access to novel ideas (25) and providing “access to scientific resources, training, expertise, and opportunities for individual and institutional capacity building and a platform for understanding global health issues and practical knowledge of how to solve complex health problems” (1). They also create enabling environments for equity and inclusion (9, 24, 26, 27). In practise, GCENs already collaborate to varying degrees, for instance through joint scientific events such as the Global Evidence Summit and coordinated initiatives such as the COVID-19 Evidence Network to support Decision-making (6). Such collaboration, however, has tended to be episodic and event-based rather than systematic, leaving substantial scope to understand how cross-network ties might be deepened.

Although extant literature has long argued that individual and organisational embeddedness within GCENs increases capacity, growth and innovation (1, 17, 23, 28), we know much less about how interactions *across* multiple networks jointly influence learning and innovation outcomes (17) or how ‘multiplexity’—the extent to which actors across networks maintain more than one type of substantive tie with each other (29)—may influence knowledge transfer, creation, and adoption. In relation to the role of GCENs in promoting and supporting evidence-based healthcare, little scholarly attention has been paid to understanding the challenges, facilitators, opportunities, or benefits of strengthening ties between and/or collaborating across GCENs.

This study focuses on the JBI Collaboration (JBIC) as its empirical setting. JBI is an international research and development organisation based in the School of Public Health at Adelaide University, Australia, that develops methodology and produces, synthesises, and translates evidence to inform health policy and practise. The JBIC is the global network of Collaborating Entities affiliated with JBI, currently comprising more than 90 entities hosted by universities, hospitals, government agencies, and professional organisations across more than 40 countries. Each entity is typically led by a Director and supported by Convenors and Deputies, and entities are established through a formal application and endorsement process against defined criteria, rather than by invitation or election. Whilst the JBIC is itself a GCEN, its members are not necessarily affiliated with other GCENs: in this study, 45% (*n* = 17) of survey respondents held membership in at least one additional GCEN (see Results), making the JBIC a useful setting for examining cross-network collaboration.

### 1.1 Objectives

The objectives of this study are to explore the experiences and views of JBI Collaboration (JBIC) members working within and across different GCENs, including the challenges, facilitators, opportunities and benefits of strengthening ties between and/or collaborating across GCENs; and to explore the opportunities for JBI and the JBIC to expand and deepen equitable collaboration with other GCENs.

## 2 Materials and methods

A 2-phase project was conducted as follows from the perspective of interpretivism using phenomenological qualitative methodology (30).

### 2.1 Phase 1: JBIC member survey

#### 2.1.1 Data collection

Phase 1: JBIC Member Survey: A brief online exploratory survey was distributed to 213 JBI Collaboration Entity Directors, Convenors, and Deputies. The survey was conducted via SurveyMonkey on October 13, 2023 (see Supplementary material 1).

#### 2.1.2 Ethics statement

This study was deemed exempt from full ethical review by the Human Research Ethics Committee (HREC) of the University of Adelaide, South Australia, Australia, in accordance with Chapter 2.1 of the National Statement on Ethical Conduct in Human Research (2007, updated 2018), on the grounds that the research presented no foreseeable risk of harm or discomfort to participants beyond minor inconvenience. However, a participant information sheet was distributed to all survey participants prior to data collection, outlining the purpose of the study, the voluntary nature of participation, the right to withdraw at any time prior to survey submission, and the anonymisation and secure storage procedures applied to all responses. Completion and submission of the survey constituted implied informed consent. For Phase 2, written confirmation of willingness to participate was obtained from all panel members, who also reviewed and validated the verbatim transcript following the session (see Supplemental material).

#### 2.1.3 Analysis

The survey comprised six closed-ended and seven open-ended questions; the full instrument is provided in Supplementary material 1. Beyond demographic and membership data (sector, country, and affiliation with other GCENs), the closed-ended items captured perceptions of open communication and knowledge sharing, opportunities for cross-network collaboration, and duplication of effort, each with a free-text elaboration. The open-ended questions explored barriers, transferable strengths of other GCENs, opportunities for collaboration, the bridging roles of individual members and leadership teams, and suggested JBI initiatives. Respondents affiliated with other GCENs answered an additional network-specific branch. Using nominal scales, closed-ended responses were analysed with descriptive statistics, cross-tabulation, and Pearson correlation coefficients (r) in Microsoft Excel; free-text elaborations were analysed as a narrative synthesis and presented alongside the quantitative findings.

To explore whether respondents’ membership in multiple GCENs was associated with their perceptions of network dynamics, Pearson correlation coefficients (equivalent to the *φ* coefficient when both variables are binary) (31) were calculated between GCEN membership (coded as 1 = member ≥1 GCENs; 0 = no other GCEN membership) and three key perceptions: opportunities for collaboration, duplication of effort, and a culture of open communication and knowledge sharing. For each perception, responses were recoded as 1 = “Yes” and 0 = “No/Unsure” to reduce sparse cells and aid interpretability. Correlations were tested two-tailed with *α* = 0.05 and are reported with df = *N*–2 (*N* = 38).

The seven open-ended questions were transcribed and analysed in Microsoft Excel, guided by Braun and Clarke’s six recursive stages of reflexive thematic analysis (32–34) (refer to thematic analysis below).

### 2.2 Phase 2: JBIC director panel

#### 2.2.1 Data collection

The panel discussion was conducted at the 70th Meeting of the JBI Collaboration on November 13, 2023. Members of the panel were a purposive sample of JBIC Entity Directors (*N* = 5) selected for their experience working with and leading groups from more than one Global Collaborative Evidence Network, with representation stratified by region, language, and World Bank income classification. Panellists represented five countries spanning three World Bank income classifications: low income (Cameroon), lower-middle income (India), and high income (USA, Finland, Czechia). Each panellist held active membership across multiple GCENs: their affiliations included JBI, Cochrane, the Africa Evidence Network, the Guidelines International Network, the GRADE Working Group, the Evidence-Based Research Network, the Evidence Synthesis Infrastructure Collaborative, and WHO Collaborating Centre networks. Their entity focus areas spanned health technology assessment, translational research, nursing and midwifery, health information and evidence standards, and clinical practise in African health systems. Because Phase 1 was distributed anonymously, it is not possible to determine whether any panellist also completed the survey. SW moderated the panel session using a question-and-answer format. The questions (see Supplementary material 2) were informed by the results of phase 1 and were circulated to panellists prior to the meeting. The 60-min panel session was transcribed verbatim from a video recording by Rev. transcription services and validated by BP and panellists.

#### 2.2.2 Thematic analysis

Thematic analysis for phases 1 and 2 was guided by the six recursive stages of reflexive thematic analysis as per Braun and Clarke (32), Braun and Clarke (33), and Braun and Clarke (34), where analysis is understood as always subjective and occurs at the intersection of the researcher(s), the data and wider contexts. Codes and themes are generated based on the researchers’ assumptions (conscious and unconscious) and interpretations of the patterns of meaning from the data, not the number of times something is said. In this regard, researcher subjectivity is deemed to benefit the analytical process, with researchers considered to play an active role in knowledge production (35). Themes were developed from responses to the seven open-ended survey questions (Supplementary material 1) and the Phase 2 panel transcript.

Coding and theme development were undertaken by three authors (BP, SW, and ZJ): BP led initial coding of both datasets, after which SW and ZJ independently reviewed the codes and candidate themes before the team refined themes collaboratively, applying this approach consistently across each analytic stage. The data for phases 1 and 2 were initially analysed as independent datasets using Microsoft Excel. Codes and supporting quotes were extracted to an Excel spreadsheet for each phase to facilitate sorting and categorisation. Initial themes were generated in an iterative process that examined codes for commonalities and moved codes in and out of categories until a pattern of codes and a set of candidate themes could be established through visual mapping and continuous engagement with the data.

The themes for each dataset from phases 1 and 2 were then analysed and grouped into four final overarching themes that were revised and reworked by the authors. Minor adaptations were made by consensus until stable themes were agreed upon by the authors. The final analysis involved defining and naming themes and selecting data excerpts that best represented each theme for a more in-depth examination.

### 2.3 Reflexivity

This study was conducted by a research team with extensive experience in evidence-based healthcare, GCEN governance, and active involvement in multiple GCENs. We acknowledge that our professional roles and affiliations positioned us as both researchers and participants in the field we were studying. This insider status provided valuable contextual understanding but also required ongoing critical reflection on how our assumptions and experiences may have shaped data interpretation.

BP and ZJ are collaborating with JBI, Cochrane, Evidence Synthesis Infrastructure Collaborative (ESIC), Guidelines International Network (GIN), Campbell Collaboration, and the World Health Organisation (WHO). DR is collaborating with JBI, Cochrane and ESIC. MK is collaborating with JBI, Cochrane, GIN, GRADE Working Group, Evidence-Based Research Network and the WHO. MPO is collaborating with JBI, Africa Evidence Network, Cochrane, GIN, WHO and ESIC. HP and KK are collaborating with JBI and WHO. SW is collaborating with JBI and Cochrane.

Following Braun and Clarke’s principles of reflexive thematic analysis, we approached coding as an active, interpretive process rather than a neutral extraction of meaning. We engaged in reflexive journaling throughout the analytic process to document our evolving thinking, interrogate our assumptions, and surface potential biases. We view our subjectivity not as a limitation but as a resource that, when critically examined, enriched the analytic depth of this study.

## 3 Results

### 3.1 JBIC member survey closed-ended questions

The survey was distributed by email to 213 JBIC Entity Directors, Convenors and Deputies (see Supplementary material 1). Of these, 19 emails failed to deliver and 194 were successfully delivered. A total of 38 responses were received, yielding a response rate of 19.6% (38 of 194 successful deliveries).

Respondents were located across 17 countries: Australia, Belgium, Brazil, Cameroon, Canada, China, Czech Republic, Ethiopia, Finland, India, Kazakhstan, Myanmar, Romania, Singapore, South Africa, UK, USA.

Most respondents (95%) identified themselves as working in the academic sector, followed by clinical settings (26%), professional societies (13%), government/policy (11%), guidelines (11%), and health technology assessment (3%). Relative to the JBIC network overall—of which approximately 77% of host institutions are academic and 29% are based in low- and middle-income countries—the sample over-represented academic respondents (95%) whilst affording somewhat stronger representation to low- and middle-income countries (42%).

The majority of respondents, 55% (*n* = 21), are not members of another Collaborative Evidence Network outside of the JBIC, whilst 20% (*n* = 10) are members of Cochrane, 10% (*n* = 5) of the Africa Evidence Network, 6% (*n* = 3) of GIN and WHO Collaborating Centres, 4% (*n* = 2) of the Campbell Collaboration and SPOR Evidence Alliance, and 2% (*n* = 1) are members of COVID-END, WHO EVIPnet and GRADE. Of these 17, nine were affiliated with one additional GCEN, five with two, two with three, and one with four. Eight (47%) were located in low- and middle-income countries, and all but one worked in the academic sector.

**Figure 1:**
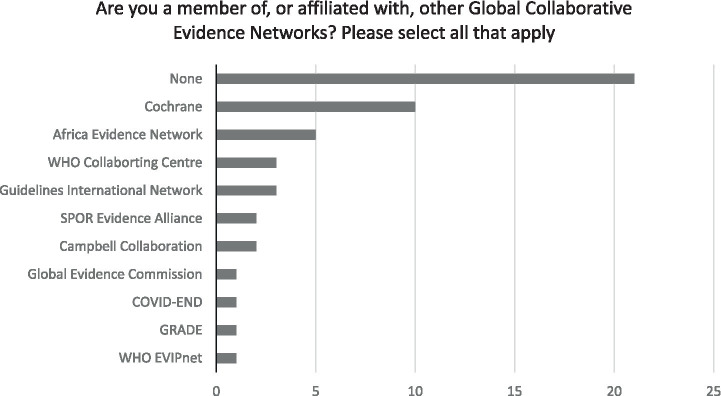
Global Collaborative Evidence Network Affiliation.

Of the 17 respondents who reported being members of other GCENs (detailed in Figure 1), 88% (*n* = 15) feel that these GCENs foster a culture of open communication and knowledge sharing across networks. Respondents attributed this to working on collaborative projects and through joint conferences/events, as well as the networks themselves sharing knowledge and communication and being “*good platforms for exchanging and sharing*.”

Three of these fifteen, however, qualified their agreement, noting that communication was “*embryonic at present*” or “*sometimes, but not effectively*” and “*yes, but in a conventional way that does perhaps not secure participation of all. Whilst they promote accessibility and inclusivity the gates to some groups and initiatives are firmly guarded by the usual suspects and those with interest are referred to less influential types of loose networks that may serve these organisations well*.”

The remaining 12% (*n* = 2) feel that these GCENs do not foster communication or knowledge-sharing across networks, noting that “*I do not feel that at present they do a great deal*—*they could do much more*” and “*No, I perceive these organisations as separate islands*.”

Across all respondents (*n* = 38), 87% (*n* = 33) feel that JBI currently fosters a culture of open communication and knowledge sharing with other GCENs, noting examples such as World Evidence-Based Healthcare Day and other joint events like the Global Evidence Summit, whilst one respondent reflected, “*I feel that this is a developing culture.*”

42% of respondents (*n* = 16) feel that there are sufficient opportunities to collaborate across GCENs, whilst 37% (*n* = 14) were unsure, and 21% (*n* = 8) feel that there are insufficient opportunities, noting that they were not aware of any opportunities, “*If these exist, I have missed them*,” and “*opportunities are not explicitly visible in communicated activities*.”

53% (*n* = 20) of respondents feel that there is a duplication of effort across GCENs, noting examples such as methods and methodologies (“*I think there is an increasing convergence of methods and interests*”; “*Method development across CENs should be consolidated*”), software for data management, and critical appraisal tools. 34% (*n* = 13) of respondents were unsure about duplication, stating that they did not know enough about the activities of other GCENs, and 13% (*n* = 5) feel like there is no duplication of effort, where “*each network seems to have their specific goals*.”

Table 1 provides a cross-tabulation of respondents’ GCEN membership with their beliefs about JBI fostering communication and knowledge sharing across GCENs, opportunities for collaboration across GCENs, and duplication of effort across GCENs.

**Table 1:** Descriptive statistics.

	GCEN membership		Do you feel that JBI fosters a culture of open communication and knowledge sharing across GCENs?			Do you feel that there are sufficient opportunities for collaboration across GCENs?			Do you feel that there is duplication of effort across GCENs?		
	Number of respondents (%)		Yes (%)	No (%)	Unsure (%)	Yes (%)	No (%)	Unsure (%)	Yes (%)	No (%)	Unsure (%)
GCEN membership	Member of other GCEN	17 (45%)	16 (94%)	0 (0%)	1 (6%)	11 (65%)	5 (29%)	1 (6%)	10 (59%)	4 (24%)	3 (18%)
	No other GCEN membership	21 (55%)	17 (81%)	0 (0%)	4 (19%)	5 (24%)	4 (19%)	12 (57%)	10 (48%)	1 (6%)	10 (48%)
	Column total	38	33	0	5	16	9	13	20	5	13

#### 3.1.1 Correlation between GCEN membership and perceptions of network functioning

There was a moderate positive correlation between GCEN membership and the belief that there are sufficient opportunities for collaboration across GCENs, *r*(36) = 0.41, 95% CI [0.11, 0.65], *p* = 0.010. A weak positive correlation was observed for perceptions that JBI fosters a culture of open communication and knowledge sharing across GCENs, *r*(36) = 0.19, 95% CI [−0.13, 0.48], *p* = 0.244. Both results should be interpreted descriptively only, given the small sample size, binary recoding of variables, and sparse cell distribution.

In contrast, correlation between GCEN membership and perceptions of duplication of effort across GCENs was very weak and non-significant, *r*(36) = 0.11, 95% CI [−0.22, 0.42], *p* = 0.505, indicating no meaningful relationship.

Taken together, these results should be treated as descriptive and hypothesis-generating only. The correlations are reported to provide a descriptive picture of the data and to identify associations worthy of investigation in future studies with larger, more diverse samples. We further note that the positive association between multi-network membership and perceiving collaboration opportunities may partly reflect that such members are already engaged in cross-network activity rather than a directional effect, reinforcing the descriptive, hypothesis-generating reading of these results.

### 3.2 Thematic analysis

Using Braun and Clarke’s reflexive thematic analysis, four interrelated themes were developed from qualitative data (34). These themes capture the challenges, opportunities, and benefits of working across GCENs (Table 2). Rather than treating collaboration as a fixed state, the analysis reveals GCENs as dynamic, relational, and situated within complex institutional and methodological ecosystems. Each theme is presented below, accompanied by illustrative participant quotes that ground the analysis in the lived realities and strategic reflections of GCEN participants. The qualitative data comprised open-ended survey responses from Phase 1—including a question branch answered only by the 17 respondents affiliated with other GCENs—together with the Phase 2 director panel transcript.

**Table 2:** Overarching themes.

Theme	Description	Key quote
Navigating barriers	Systemic, operational and epistemic frictions (disparate KPIs/reporting, time and funding constraints, duplicated methods, uneven access) impede cross-network collaboration and communication.	“There are various requirements for reporting to KPIs, outputs, et cetera… when you are in a time and money pressure … it does require … unfortunate prioritisation.”
Fostering synergy	Boundary-spanning members broker ties; trust, reciprocity, and co-design, often strengthened by face-to-face contact, enable information sharing and joint work.	“Physical interaction through meetings will enable more sharing across networks because of increased trust and commitments.”
Benefits of ties	Multiplex membership amplifies reach and legitimacy, combines complementary capacities, and supports impact (from national policy influence to crisis response).	“Having a collaboration with WHO, JBI… we were able to save lives.”
Harmonisation opportunities	Strategic alignment through shared infrastructures, method co-development, and clearer pathways/umbrella arrangements can reduce duplication and increase coherence.	“We do not need more international organisations. We need the one which will be effectively connecting all we have.”

#### 3.2.1 Theme 1: navigating organisational, methodological, and resource barriers

Participants described a range of systemic, operational, and epistemological barriers that inhibit deeper collaboration across GCENs. These included fragmented governance systems, inconsistent performance reporting requirements, and duplicated methodological efforts. Respondents emphasised that these structural frictions often resulted in disjointed priorities, diluted efforts, and fatigue.

“There are various requirements for reporting to KPIs, outputs, et cetera, to each of the individual entities … when you are in a time and money pressure … it does require … unfortunate prioritisation.”

Time and funding constraints were cited as key obstacles, especially for those engaged in multiple networks or located in resource-constrained settings. Limited staffing and the need to meet diverse network demands often placed a significant burden on institutions and individuals. In effect, each additional network multiplied the time demanded for events and reporting, so that where staffing was limited this burden concentrated on the same few individuals. As one Phase 2 panellist described:

“If you are on many networks, you now have to … be at the different conferences, to also provide reports to the different networks. … So, one of our biggest challenges was staffing.”

Beyond resource pressures, participants pointed to methodological tensions. Variability in philosophical paradigms, evidence standards, and operational procedures created difficulties for alignment. Whilst some welcomed pluralism, others called for strategic harmonisation to reduce inefficiencies.

“Each GCEN has their respective methodologies … harmonisation or choosing to focus on a limited set of methodologies … bears discussion.”

Power dynamics also surfaced as a barrier, particularly in the strategic governance layer of networks. Participants expressed concern that decision-making could become politicised, sidelining substantive intellectual collaboration and constraining innovation.

“The strategic management level has become so political … the politics have come to hinder much of the early intellectual work.”

#### 3.2.2 Theme 2: fostering synergy, collaboration, and knowledge sharing

Despite these challenges, participants articulated a strong belief in the potential of cross-network collaboration to foster innovation, reduce duplication, and extend reach. Many saw themselves as ‘boundary spanners’—performing what the brokerage literature would term a bridging role—leveraging dual or multiple memberships to facilitate coordination and mutual learning.

“We’ve really been trying to be almost a mediator … to bridge the gap around resource and resource waste.”

Trust and reciprocity were recurrently cited as essential conditions for effective collaboration. Participants emphasised the value of face-to-face interaction and sustained dialogue to build relationships that transcend institutional mandates.

“Physical interaction through meetings will enable more sharing across networks because of increased trust and commitments.”

Co-design and co-contribution emerged as key practises underpinning successful collaboration. Participants described a willingness to work collectively, highlighting the significance of shared goals and aligned missions.

“There is a willingness to work together … I find people from different networks are happy to contribute.”

Importantly, this collaborative culture was often championed and maintained by individuals rather than systems, underscoring the relational labour required to activate synergies across complex networks. Trust was built incrementally—through repeated face-to-face contact, joint projects, and reciprocal exchange—and because this work rested on individuals, participants saw scope for networks to support it systemically, for example by creating structured opportunities for interaction and formally recognising those who take on bridging or leadership roles. Participants also emphasised that trust of this kind is inherently time-dependent: it accrues through sustained engagement over months and years rather than through one-off interactions, making the long-term continuity of GCEN membership itself a structural asset for collaboration.

#### 3.2.3 Theme 3: having ties with multiple GCENs yields benefits

A significant strength of GCENs, participants argued, lies in their capacity to amplify impact. Working across multiple networks enabled actors to combine technical resources, influence policy at national and international levels, and build collective credibility.

“Having a collaboration with WHO, JBI and others … we were able to save lives … That is a model where we can show what was the result of this collaboration.”

In addition, GCENs were seen as enablers of South–South collaboration, allowing institutions in LMICs to exchange contextualised strategies and innovations, bypassing traditionally Northern-centric hierarchies of evidence dissemination. One panellist described how their Indian centre, a WHO Collaborating Centre, partnered to respond to a request from Somalia during the COVID-19 pandemic:

“So, during COVID, there was a call from Somalia, they did not have electricity in their healthcare Centres… we need to develop an oxygen concentrator with solar energy…. But that was only possible when we had a collaboration and we had different centres, dedicated centres for technology… and building an evidence around all these innovations, process modifications and everything. So, that is an immense potential… and this was South–South collaboration.”

Membership across several networks enhanced legitimacy, visibility, and political capital, particularly in policy settings where alignment with global standards was beneficial.

“Taking part in these different networks … the politicians also see us as more valuable for the national purpose.”

Participants also highlighted the pragmatic value of diversity within GCENs. Different networks brought complementary methods, capacities, and tools that could be mobilised in response to distinct challenges.

“Various needs also need various resources. And that’s why we find ourselves in so many collaborations.”

Some respondents noted that network participation enabled access to international research funding, tools, and policy platforms, especially when local systems were underdeveloped or lacked legitimacy.

“In our country, national institutions do not always trust local research unless it’s been done with WHO or another big name. So being part of the GCENs has helped us get our work taken seriously.”

Others highlighted the symbolic value of GCEN involvement in motivating teams and demonstrating global alignment, which helped strengthen institutional commitment to evidence-based practise.

“The visibility and recognition we get by being in different GCENs gives us more leverage with our university leadership to prioritise evidence-based healthcare.”

The benefits of cross-network engagement were not merely instrumental. Participants described increased confidence, capability building, and pride in contributing to a broader global evidence ecosystem, suggesting that collaboration across GCENs fosters not just impact but community.

Participants also described the role of cross-GCEN engagement in building methodological competencies, particularly in evidence synthesis, critical appraisal, and implementation science, not widely available through institutional channels alone. One panellist from Cameroon described deliberately engaging younger team members in cross-network activities, noting that “moving this forward is only possible if her capacity is built—and this cuts across different networks.” Survey respondents similarly called for aligned training opportunities across GCENs, citing workshops in evidence synthesis methods as a way to develop skills whilst reducing duplication of effort. Critically, however, one panellist cautioned that capacity building can inadvertently deepen inequity if not designed with end-users in mind: a JBI audit and feedback intervention in malaria treatment had increased the knowledge gap between clinicians and policymakers, illustrating that well-intended capacity building can create new asymmetries. This points to a fundamental equity imperative: cross-GCEN capacity building must be designed to reach institutions and individuals most constrained by existing knowledge hierarchies, not only those already positioned to benefit.

“In addition we have richer experience, projects, publications and other output in EBHC because of participation in different networks, that made us feel confidence in international EBP family.”

“What I see in this growing collaboration between the collaborative evidence networks is the ability for us to meet that goal of evidence-based healthcare really in a broader way and not just meet the specific outcomes for each organisation, but truly create the global impact that we are all after.”

#### 3.2.4 Theme 4: opportunities for harmonisation across GCENs: leveraging existing strengths, finding synergies

Looking forward, participants expressed optimism about the potential to integrate and strategically align GCENs. Rather than proliferating new structures, they advocated for leveraging existing networks more effectively through shared infrastructure, membership models, and common reporting frameworks. As one Phase 1 respondent put it directly:

“We do not need more international organisations. We need the one which will be effectively connecting all we have.”

Calls for harmonisation were not limited to systems but extended to ethos and practise. Participants emphasised the need for stronger participatory mechanisms and co-produced outputs that centre the needs of end-users and historically marginalised actors.

“What we do not do, in my assessment, across any of the networks, is a good enough job engaging those that are ultimately impacted by the work we produce.”

There was enthusiasm for uniting efforts in methodological development, an area viewed as both fragmented and foundational to collaboration.

“There’s a lot of opportunity around methods and methodology … I think the inclusive nature of JBI really positions it to be the leader of helping those collaborations.”

“GCEN entities may consider either harmonising methodologies and methods or each GCEN select a set of methodologies and methods as their focus (reduce duplication of effort).”

Some participants proposed formal mechanisms such as shared membership frameworks, interoperable databases, and aligned capacity-building programmes as ways to reduce administrative burden and increase access.

“Maybe if the networks could align how they do capacity building, we could train people once and then plug them into several streams of activity. It would save time and bring more coherence.”

Others saw value in coordinated governance to clarify roles, reduce competition, and develop joint strategies.

“We need some kind of governance mechanism—not to control, but to guide. Something that helps us navigate overlaps and decide what each network is best placed to lead.”

## 4 Discussion

This study offers exploratory empirical insights into how collaboration across GCENs is experienced in practise, drawing on perspectives from JBIC-recruited participants, a significant proportion of whom held concurrent membership across multiple GCENs, and extending the literature on networks, knowledge exchange, and multiplexity. Given the modest sample size and the JBIC recruitment frame, the findings should be understood as exploratory and hypothesis-generating rather than broadly generalisable, and future comparative research across additional GCENs is warranted. Within these bounds, the results reaffirm the complex, relational nature of GCENs whilst illustrating how cross-network engagement can generate innovation, foster system resilience, and amplify collective impact. These findings both support and extend prior research whilst highlighting gaps and opportunities for future coordination for networks across the global evidence ecosystem.

### 4.1 Building bridges between networks: brokers and bridging

Connections between groups, what network theorists call “bridging ties,” play a vital role in enabling cross-network learning, innovation, and resilience (20, 36). The diffusion of novel information across these bridges supports what Granovetter (37) and Granovetter (38) described as the “strength of weak ties”: the idea that links between otherwise unconnected actors or groups offer access to knowledge and resources that are unavailable within tightly knit communities. In the context of GCENs, this bridging function was repeatedly emphasised by participants who described how individuals affiliated with multiple networks serve as conduits for ideas, tools, and opportunities. As one Phase 2 panellist observed, “There are lots of silos, if you are not part of one network, you might miss out. Having people straddling both means things do not fall through the cracks.” A Phase 1 survey respondent similarly emphasised, “We’ve really been trying to be almost a mediator … to bridge the gap around resource and resource waste.” Phase 1 open-ended responses reinforced this picture, with one respondent calling for JBI to “build lots of bridges to link up organisations”—suggesting awareness that bridging structural holes requires not just willing individuals but deliberate institutional investment. The ability of bridges between networks to diffuse diverse information becomes significant when that information is important and time-sensitive, such as COVID-19. The need for such bridges becomes even more acute in LMIC contexts, where institutional capacity is still maturing, and brokering roles can catalyse not just exchange, but actual systems development.

Phase 1 survey data further support this view, with over 85% of all respondents agreeing that greater coordination between GCENs is essential to reduce duplication and improve efficiency. However, fewer than half of all respondents reported having formal mechanisms for cross-network communication, highlighting the reliance on informal relationships and individual actors to bridge structural holes. Across both phases, participants positioned themselves as brokers—those actors who span boundaries and enable interaction across disconnected domains—navigating across GCENs to mediate, share, and mobilise diverse forms of expertise. This reflects the relational labour inherent in building and sustaining multiplex ties (29, 39), which enable actors to move between institutional contexts whilst maintaining shared purpose. As Long et al. (40) and Kwon et al. (41) have shown, such individuals are not only conduits of information but also translators of language, norms, and institutional logics. Participants in this study frequently described this type of translational work. One respondent explained, “I often find myself explaining one network’s methods or acronyms to members of another. It’s not just about sharing information, it’s about making it make sense.” Importantly, brokers wield their influence between groups, rather than within them. This means that brokers can help fill structural holes or bridge networks that do not overlap. They help to connect at least two networks and therefore can create bridging social capital, including access to information or other resources not already available in one’s network. However, brokers in GCENs do more than link institutions, they also connect knowledge paradigms, value systems, and geographies. In this study, participants highlighted the importance of such translational figures in making global networks feel locally relevant. As one participant shared, “We do a lot of translation work across networks. We do not just translate documents, we translate intent, so that what’s global resonates in our national context.” This aligns with Bräuchler et al. (42), who describe brokers as “situated between various social worlds… translating different languages and jargons” and thereby constituting the very relationships they mediate. This is especially relevant in efforts to localise global guidance on evidence-based healthcare in LMICs, where frameworks developed in high-income countries often require adaptation through culturally and policy-driven translation.

Building bridges also means structurally recognising LMIC-led innovations and integrating them as central, not peripheral, elements in the global evidence ecosystem.

Bridging also creates access to symbolic capital. Being visibly embedded in multiple GCENs carries reputational advantages that can help local organisations or units gain credibility with funders and policymakers. This was reflected in both phases of the study. Phase 1 survey respondents frequently cited enhanced visibility and credibility as key benefits of GCEN involvement. As one survey respondent explained, “We’ve used our connections across networks to strengthen our funding applications, as we can show we are trusted by multiple global partners.”

The participants’ descriptions of mediating across disconnected networks—linking institutions, translating knowledge systems, and facilitating cross-boundary exchange—map directly onto what the brokerage literature identifies as its defining functions. Building on this, the analysis introduces further conceptual nuance by distinguishing between two forms of brokerage evident in the data. Finally, bridging GCENs is not only structural but also deeply cultural. This study introduces conceptual nuance by distinguishing between structural brokerage, linking disconnected institutional or functional nodes, and *transcultural brokerage*, which involves navigating across epistemic, linguistic, and geographic divides. Transcultural brokers emerged as key actors in enabling inclusive and context-sensitive collaboration. As one participant observed, “And in all these three roles [across GCENs], what we do, we promote evidence-based nursing and also midwifery from different perspectives. So, we are doing a lot from different perspectives, but I think this all gives synergy,” underscoring the importance of integrating diverse worldviews and practises. This aligns with Levy et al. (16), who describe cosmopolitan actors as those capable of bridging both structural and cultural holes, operating across knowledge paradigms and cultural contexts to facilitate equitable global collaborations.

A noteworthy finding from Phase 2 is the recognition that GCENs are often still perceived as “separate islands.” Efforts to bridge these islands require intentional design and policy support. Participants emphasised the value of shared events, cross-membership, and dedicated brokerage roles to institutionalise linkages. As one participant suggested, “Perhaps we need an umbrella organisation which will be effectively connecting all we have.” This highlights the importance of *institutionalising brokerage*, rather than relying solely on individual actors. Whilst informal brokers are invaluable, their roles are often unrecognised and unsupported. As one respondent noted, “We need more formal structures, otherwise, we are dependent on personalities, and that’s not sustainable.” Phase 1 responses echoed this sentiment, with calls for structured roles, joint initiatives, and shared platforms to support ongoing collaboration.

### 4.2 Communication as collaboration: building the conditions for trust, reciprocity, and coordination

Effective collaboration across GCENs is contingent upon a culture of intentional, inclusive, and ongoing communication. Whilst many participants described a strong interpersonal commitment to sharing, there was widespread acknowledgement that systemic communication remains underdeveloped. One respondent described the challenge as “infrequent communications and very passive approach to collaboration across networks,” whilst another noted, “I just find everyone is still working in silos.”

In Phase 1, 88% of respondents affiliated with more than one GCEN agreed that these networks foster a culture of open communication and knowledge sharing, often through collaborative projects, joint events, and informal exchanges. However, these positive reflections were tempered by concerns about formality and access. Several respondents noted that this communicative culture was “embryonic at present” or “not effective,” with one warning that “the gates to some groups and initiatives are firmly guarded by the usual suspects.” Others were more sceptical, characterising networks as “separate islands” and calling for more inclusive, structured forms of engagement.

Participants consistently stressed the importance of both formal and informal channels of communication. As one panellist reflected, “Communication is the core of the processes through which collaboration takes place… it must be regular, active, reciprocal and open.” Another emphasised that collaboration is sustained not only through ‘formal communications’, i.e., team meetings and projects, but also “informal communication (in-person events, lunchtime chats and email exchanges).” These findings reinforce the idea that communication is not merely about exchanging information, but also about building relational trust and mutual accountability over time.

The relational dimensions of communication were particularly evident in discussions of face-to-face interaction. Several participants affirmed that physical meetings, conferences, and working groups are critical in building trust and reinforcing commitments. One respondent shared, “When people meet physically, they tend to be more reassured and encouraged, this builds trust.” Events such as the Global Evidence Summit and World Evidence-Based Healthcare Day were frequently cited as important vehicles for breaking down silos and reinforcing a shared identity across GCENs. As one participant noted, “The first Global Evidence Summit, the JBI GIN conference… these are all very good examples [of joint communication in action].”

The study also revealed, however, significant gaps in communication infrastructure. Only 42% of survey respondents felt that there are sufficient opportunities to collaborate across GCENs, and many indicated that opportunities were not well publicised or accessible. One participant remarked, “If these exist, I have missed them,” whilst another noted that “opportunities are not explicitly visible in communicated activities.”

Taken together, these findings suggest that whilst individual commitment and network-level goodwill exist, the enabling conditions for effective communication are not yet fully institutionalised. What is needed is not only more communication, but better communication: investments in communication infrastructure, structured platforms, clearer entry points, and shared language that allows diverse actors to engage equitably.

### 4.3 Amplifying global impact through cross-network engagement

This study affirms that cross-network engagement with GCENs serves not only instrumental but also symbolic functions. Participants consistently described how involvement in multiple GCENs increased their institutional visibility, policy influence, and perceived legitimacy. In both high-resource and resource-constrained contexts, GCEN affiliations were seen as mechanisms to gain recognition, access international funding, and leverage broader platforms for influence. As one participant observed, “Taking part in these different networks … the politicians also see us more valuable for the national purpose.”

These insights align with Stewart’s (23) argument that evidence networks enable local actors to “speak globally” whilst acting locally, thereby enhancing their credibility within national systems through their connection to global standards and partnerships. The findings further reveal that GCEN engagement enhances not only political capital but also team morale, institutional commitment to evidence-based practise, and the ability to act during global health emergencies. As one participant recounted, “Having a collaboration with WHO, JBI and others … we were able to save lives.”

Importantly, participants also highlighted the pragmatic value of working across multiple GCENs, which provided access to complementary capacities, ranging from methods and resources to synthesis tools and implementation strategies. This strategic diversity allowed institutions to respond to complex challenges with agility. As one panellist explained, “Various needs also need various resources. And that’s why we find ourselves in so many collaborations.”

Yet the symbolic capital afforded by GCEN affiliation is not evenly distributed. Several participants noted that legitimacy often accrues more readily to institutions with longstanding ties to high-profile or Northern-based networks, reinforcing global hierarchies. In some contexts, affiliation with a recognised GCEN was essential to ensure local research was taken seriously: “In our country, national institutions do not always trust local research unless it’s been done with WHO or another big name.” These dynamics echo the critique by Oliver et al. (9), who warn that symbolic legitimacy can become concentrated amongst already dominant actors, further marginalising others. There is thus a critical need for mechanisms within GCENs to intentionally elevate underrepresented voices, support capacity-building in emerging research settings, and co-create knowledge outputs that reflect regional priorities.

The study also surfaced a performative dimension to GCEN membership. Phase 2 panellists and Phase 1 survey respondents alike described how affiliations were highlighted strategically in grant applications, policy dialogues, and institutional communications to enhance legitimacy and credibility. This suggests that GCENs function as both relational and symbolic infrastructures, conferring status and authority that may, at times, be more visible than substantive. As a Phase 1 survey respondent reflected, “The visibility and recognition we get by being in different GCENs gives us more leverage with our university leadership to prioritise evidence-based healthcare.” These findings underscore the need for collaborative mechanisms that are sensitive to power asymmetries and committed to redistributing the benefits of symbolic capital.

### 4.4 Tensions, trade-offs, and transformative potential

Whilst participants consistently recognised the value of cross-network engagement, they also identified a range of persistent barriers that complicate collaboration. These include structural, methodological, and political tensions that reflect the broader paradox of ‘coopetition’, where cooperation and competition must be navigated simultaneously (43–45). For example, the need for equitable authorship and data ownership policies to address power imbalances in collaborative research outputs.

Resource constraints featured prominently across both phases. Working across multiple GCENs demands significant time, staffing, and financial commitment, especially when organisations must contribute to overlapping events, reporting requirements, and governance systems. One panellist explained, “You now have to … be at the different conferences, to also provide reports to the different networks … staffing was a challenge.” Others echoed the difficulty of managing conflicting expectations across networks that lack coordinated infrastructure or recognition systems. As one Phase 1 survey respondent put it, “What may give credit in standing with Cochrane is not recognised within a JBI matrix.”

Participants also raised concerns about methodological duplication. Many expressed frustrations at the proliferation of software tools, evidence standards, and review methodologies across GCENs, noting that “everyone has software for data management, tools for appraisal, would be nice to have an agreed system.” At the same time, participants cautioned against over-standardisation. As one observed, “Each GCEN has their respective methodologies … harmonisation or choosing to focus on a limited set of methodologies … bears discussion.” Calls for strategic harmonisation across GCENs echo Stewart's (23) argument that networks can achieve greater impact when aligned around shared infrastructures and priorities. These findings support a pluralistic yet strategically aligned approach, one that preserves methodological diversity whilst fostering transparency, coordination, and shared goals.

Political dynamics at the strategic level of networks also emerged as a key challenge. Phase 2 panellists, in particular, described how governance environments can become overly politicised, suppressing intellectual collaboration and marginalising dissenting perspectives. One panellist reflected, “The politics have come to hinder much of the early intellectual work. The general tendency is: if you are not part of the mainstream or if you have a dissonant voice, move aside.” Such dynamics underscore the importance of governance and collaboration mechanisms that safeguard diversity, actively engage underrepresented actors, and foster spaces for networks to negotiate roles and responsibilities, rather than compete for them. This is particularly salient in the context of institutionalised inequalities within GCENs: when networks are perceived as dominated by Western institutions, their external legitimacy in diverse policy contexts is weakened, further entrenching structural hierarchies (46).

Together, these tensions highlight the transformative potential for GCENs to coordinate more deliberately whilst respecting the distinct contributions of each. Rather than seeking homogeneity, the path forward lies in designing mechanisms that enable transparency, strategic alignment, mutual recognition, and equitable collaboration. Realising this potential will require concrete structural commitments, i.e., governance frameworks that distribute decision-making authority beyond well-resourced Northern entities; pooled or subsidised funding mechanisms that do not require under-resourced partners to compete on equal terms; co-designed reporting systems that reduce the disproportionate administrative burden borne by Southern collaborators; and explicit equity benchmarks embedded in collaborative frameworks from the outset.

### 4.5 Equity as a strategic value for GCENs—a challenge yet a northern star

Equity is a strategic value for network architectures, GCENs must redistribute symbolic, relational, and material capital including capacity sharing and mutual understanding of challenges across contexts. There is also a need for institutional mechanisms that support equity, such as pooled funding, transparent equitable authorship norms (10, 47), and visibility platforms for LMICs. Some ideas that could facilitate equity include shared funding mechanisms accessible to LMICs, formal recognition systems for brokers from LMICs, network-wide equity audits and dashboards, and LMIC-led methods hubs or working groups. Although this is challenging, it must remain the Northern Star for GCENs because equity is essential to both their legitimacy and sustainability.

### 4.6 Contributions to the literature, limitations, and future research directions

This study makes several novel contributions. First, it provides empirical support for the value of multiplexity across GCENs, highlighting both its potential and its limits when unsupported by formal structures. Second, it highlights the relational, political, and institutional labour of collaboration (brokering), complementing models of network function with a more human-centred understanding. Third, it contributes to a growing literature on network sustainability, offering a vision of strategic integration grounded in equity, trust, and reflexivity.

This study should, however, be considered in light of its limitations. First, the Phase 1 survey had a modest response rate, which may limit the generalisability of findings across the broader JBIC and GCEN landscape. The sample was predominantly academic (95%), over-representing this sector relative to the broader network, and analyses concerning cross-network membership rest on the 17 respondents affiliated with other GCENs, approximately 8% of those invited, further constraining representativeness. Second, whilst participants were recruited through JBIC, a significant proportion held active membership across multiple GCENs, lending cross-network experiential validity to the findings. Nonetheless, the recruitment frame and modest sample size mean that findings should be understood as exploratory and hypothesis-generating rather than representative of the broader GCEN landscape. The Phase 2 panel was also a purposive sample of five individuals, and whilst selected for their multi-network experience, their perspectives should not be extrapolated as definitive. Future comparative investigation recruiting directly across multiple GCENs would meaningfully extend this work. Third, this study is subject to insider positionality: the research team are themselves members of JBI and the JBIC network, and the Phase 1 survey was distributed to a closed professional community of which the authors form part. This proximity, whilst enabling access and contextual depth, introduces the potential for social desirability bias, with participants potentially moderating their responses in awareness of the relational context. Fourth, the Phase 2 panel was purposively rather than randomly constituted, and panellists’ perspectives feature prominently across the thematic analysis and direct quotation. Whilst this reflects the depth and range of experience they contributed, it also means their views may carry disproportionate weight in shaping the analytic narrative, and findings should be read with this in mind. Future studies would benefit from expanding participation and triangulating findings across additional GCENs.

Future research should also explore the institutional conditions that allow network brokers to thrive and develop comparative models of GCEN governance that align diversity with shared values. Further investigation is needed into how end-users—practitioners, communities, and policymakers—can be more meaningfully integrated into the collaborative governance of GCENs (5, 48). Additionally, the role of digital platforms and technologies in enabling cross-network collaboration remains under-examined and warrants deeper investigation.

Ultimately, this study supports the call for GCENs to evolve from siloed, often fragmented entities into a more coherent, adaptive ecosystem, which will require sustained investment in relational and institutional infrastructure.

## 5 Conclusion: reimagining GCEN collaboration

This study provides exploratory evidence pointing towards the potential value of moving from fragmented coexistence to a more strategic and integrated model of collaboration across GCENs. Whilst participants widely recognised the value of working across networks, they also highlighted that collaboration remains *ad hoc*, under-resourced, and overly dependent on individual brokers. Respondents described a landscape marked by duplicated efforts, uncoordinated initiatives, and opaque opportunities, with some likening GCENs to “separate islands.”

Rather than advocating for consolidation or uniformity, participants envisioned a model of purposeful convergence, one grounded in complementarity, shared purpose, and mutual respect for the distinct strengths of each network. Multiplex engagement, where individuals navigate multiple GCENs simultaneously, has been shown to generate cross-pollination, enhance legitimacy, and broaden access to global resources. Yet this bridging work often goes unrecognised institutionally, highlighting the need to formalise and support relational labour through structural mechanisms.

The findings point towards the need for systemic solutions that enable collaboration. Proposed innovations included interoperable platforms for protocol registration and data sharing, aligned capacity-building efforts, shared events, and harmonised reporting systems. These practical steps were seen as ways to reduce duplication, improve coherence, and build trust. Equally important were calls for governance models that are participatory and equity-sensitive, recognising disparities in access, visibility, and influence across networks and geographies.

Underlying these proposals is a shift in how collaboration is conceptualised: not simply as episodic cooperation, but as an ongoing, institutionally embedded practise. Future GCEN collaboration will need to be underpinned by intentional design, shared infrastructures, and co-created frameworks that support joint agenda-setting, resource mobilisation, and inclusive engagement. Yet, to fully realise this value, there is a pressing need to move from informal coordination to structured, intentional collaboration mechanisms.

By institutionalising these collaborative practises and addressing power asymmetries, GCENs may be better positioned to generate and translate evidence for equitable global health impact. The challenge now is to reimagine collaboration not as an aspiration, but as a sustained mode of operating—one that reflects the complexity, diversity, and interdependence of the global evidence ecosystem.

## Data Availability

The original contributions presented in the study are included in the article/Supplementary material, further inquiries can be directed to the corresponding author.

## References

[1] Pilla B Jordan Z Christian R Kynoch K Mcinerney P Cooper K . JBI series paper 4: the role of collaborative evidence networks in promoting and supporting evidence-based health care globally: reflections from 25 years across 38 countries. J Clin Epidemiol. (2022) 150:210–5. doi: 10.1016/j.jclinepi.2022.04.009, 35462046

[2] Camarinha-Matos LM Afsarmanesh H. Collaborative Networks. International Conference on Programming Languages for Manufacturing. Boston (MA): Springer (2006). p. 26–40.

[3] Jordan Z Pilla B. From agenda to action: JBI evidence synthesis and the United Nations sustainable development goals. JBI Evid. Synth. (2024) 22:364–77. doi: 10.11124/JBIES-23-00088, 37851334

[4] Pilla B Stone J Jordan Z. Cracking the code: Ai’s role in mapping evidence syntheses to the United Nations sustainable development goals. Arch Public Health. (2025) 83:318. doi: 10.1186/s13690-025-01784-0 41291944 PMC12751934

[5] Lavis J. Global Commission on Evidence to Address Societal Challenges-10 March 2022. Hamilton (ON): McMaster Health Forum (2022).

[6] Mccaul M Tovey D Young T Welch V Dewidar O Goetghebeur M . Resources supporting trustworthy, rapid and equitable evidence synthesis and guideline development: results from the Covid-19 evidence network to support decision-making (Covid-end). J Clin Epidemiol. (2022) 151:88–95. doi: 10.1016/j.jclinepi.2022.07.008, 35868494 PMC9295316

[7] Pilla B Porritt K Jordan Z. Addressing global health equity through global collaborative evidence networks: a narrative literature review of governance models, power and participation. Glob Health. (2026) 22:28. doi: 10.1186/s12992-026-01192-1, 41654843 PMC12977543

[8] Matenga TFL Zulu JM Corbin JH Mweemba O. Dismantling historical power inequality through authentic health research collaboration: southern partners’ aspirations. Glob Public Health. (2021) 16:48–59. doi: 10.1080/17441692.2020.1775869, 32496873

[9] Oliver S Bangpan M Stansfield C Stewart R. Capacity for conducting systematic reviews in low-and middle-income countries: a rapid appraisal. Health Res Policy Syst. (2015) 13:1–8. doi: 10.1186/s12961-015-0012-0, 25928625 PMC4443541

[10] Gautier L Sieleunou I Kalolo A. Deconstructing the notion of “global health research partnerships” across northern and African contexts. BMC Med Ethics. (2018) 19:49–20. doi: 10.1186/s12910-018-0280-7, 29945595 PMC6019997

[11] Matenga TFL Zulu JM Corbin JH Mweemba O. Contemporary issues in north–south health research partnerships: perspectives of health research stakeholders in Zambia. Health Res Policy Syst. (2019) 17:1–13. doi: 10.1186/s12961-018-0409-7, 30646902 PMC6334387

[12] Kelaher M Ng L Knight K Rahadi A. Equity in global health research in the new millennium: trends in first-authorship for randomized controlled trials among low-and middle-income country researchers 1990-2013. Int J Epidemiol. (2016) 45:2174–83. doi: 10.1093/ije/dyw313, 28199646

[13] Akerlof K Timm K Chase A Cloyd ET Heath E Mcghghy B . What does equitable co-production entail? Three perspectives. Community Sci. (2023) 2:e2022CSJ000021. doi: 10.1029/2022CSJ000021, 41816204 PMC12973253

[14] Turnhout E Metze T Wyborn C Klenk N Louder E. The politics of co-production: participation, power, and transformation. Curr Opin Environ Sustain. (2020) 42:15–21. doi: 10.1016/j.cosust.2019.11.009

[15] Kumar R Khosla R Mccoy D. Decolonising global health research: shifting power for transformative change. Plos Global Public Health. (2024) 4:e0003141. doi: 10.1371/journal.pgph.0003141, 38656955 PMC11042701

[16] Levy O Lee H-J Jonsen K Peiperl MA. Transcultural brokerage: the role of cosmopolitans in bridging structural and cultural holes. J Manag. (2019) 45:417–50. doi: 10.1177/0149206318773404

[17] Phelps C Heidl R Wadhwa A. Knowledge, networks, and knowledge networks: a review and research agenda. J Manag. (2012) 38:1115–66. doi: 10.1177/0149206311432640

[18] Bouty I. Interpersonal and interaction influences on informal resource exchanges between R&D researchers across organizational boundaries. Acad Manag J. (2000) 43:50–65. doi: 10.2307/1556385

[19] Hemphälä J Magnusson M. Networks for innovation–but what networks and what innovation? Creat Innov Manag. (2012) 21:3–16. doi: 10.1111/j.1467-8691.2012.00625.x

[20] Tortoriello M Reagans R Mcevily B. Bridging the knowledge gap: the influence of strong ties, network cohesion, and network range on the transfer of knowledge between organizational units. Organ Sci. (2012) 23:1024–39. doi: 10.1287/orsc.1110.0688, 19642375

[21] Rafique T Mahmood S Butt FS Mehmood T. The mediating role of absorptive capacity and learning networks in the relationship between inter-organizational learning and innovation capability. J Posit Sch Psychol. (2022) 6:1917–27.

[22] Back A Von Krogh G Seufert A Enkel E. Putting Knowledge Networks Into Action: Methodology, Development, Maintenance. Berlin: Springer (2005).

[23] Stewart R. Do evidence networks make a difference? J Dev Eff. (2018) 10:171–8. doi: 10.1080/19439342.2018.1425734

[24] Stewart R Dayal H Langer L Van Rooyen C. The evidence ecosystem in South Africa: growing resilience and institutionalisation of evidence use. Palgrave Commun. (2019) 5:1–12. doi: 10.1057/s41599-019-0303-0

[25] Battistella C De Toni AF Pillon R. Inter-organisational technology/knowledge transfer: a framework from critical literature review. J Technol Transf. (2016) 41:1195–234. doi: 10.1007/s10961-015-9418-7

[26] Abimbola S. The Foreign Gaze: Authorship in Academic Global Health. London: BMJ Publishing Group Ltd (2019). 10.1136/bmjgh-2019-002068 PMC6830280 31750005

[27] Chu KM Jayaraman S Kyamanywa P Ntakiyiruta G. Building research capacity in Africa: equity and global health collaborations. PLoS Med. (2014) 11:e1001612. doi: 10.1371/journal.pmed.1001612, 24618823 PMC3949667

[28] Shipilov A Li SX Bothner MS Truong N. Network advantage: uncontested structural holes and organizational performance in market crises. Strateg Manag J. (2023) 44:3122–54. doi: 10.1002/smj.3538

[29] Lazega E Pattison PE. Multiplexity, generalized exchange and cooperation in organizations: a case study. Soc Networks. (1999) 21:67–90. doi: 10.1016/s0378-8733(99)00002-7

[30] Lincoln Y Guba B. Naturalistic Inquiry. Beverly Hills: Sage Publikations. Inc. (1985).

[31] Sedgwick P. Pearson’s correlation coefficient. BMJ. (2012) 345:e4483–3. doi: 10.1136/bmj.e4483

[32] Braun V Clarke V. Using thematic analysis in psychology. Qual Res Psychol. (2006) 3:77–101. doi: 10.1191/1478088706qp063oa

[33] Braun V Clarke V. Can I use ta? Should I use ta? Should I not use ta? Comparing reflexive thematic analysis and other pattern-based qualitative analytic approaches. Couns Psychother Res. (2021) 21:37–47. doi: 10.1002/capr.12360

[34] Braun V Clarke V. Conceptual and design thinking for thematic analysis. Qual Psychol. (2022) 9:3–26. doi: 10.1037/qup0000196

[35] Braun V Clarke V. One size fits all? What counts as quality practice in (reflexive) thematic analysis? Qual Res Psychol. (2021) 18:328–52. doi: 10.1080/14780887.2020.1769238

[36] Tiwana A. Do bridging ties complement strong ties? An empirical examination of alliance ambidexterity. Strateg Manag J. (2008) 29:251–72. doi: 10.1002/smj.666

[37] Granovetter M. The strength of weak ties: a network theory revisited. Sociol Theory. (1983) 1:201–33. doi: 10.2307/202051

[38] Granovetter MS. The strength of weak ties. Am J Sociol. (1973) 78:1360–80. doi: 10.1086/225469

[39] Wang R Tanjasiri SP Palmer P Valente TW. Network structure, multiplexity, and evolution as influences on community-based participatory interventions. J Community Psychol. (2016) 44:781–98. doi: 10.1002/jcop.21801, 29430067 PMC5807015

[40] Long JC Cunningham FC Braithwaite J. Bridges, brokers and boundary spanners in collaborative networks: a systematic review. BMC Health Serv Res. (2013) 13:1–13. doi: 10.1186/1472-6963-13-158, 23631517 PMC3648408

[41] Kwon S-W Rondi E Levin DZ De Massis A Brass DJ. Network brokerage: an integrative review and future research agenda. J Manag. (2020) 46:1092–120. doi: 10.1177/0149206320914694

[42] Bräuchler B Knodel K Röschenthaler U. Brokerage from within: a conceptual framework. Cult Dyn. (2021) 33:281–97. doi: 10.1177/09213740211011202

[43] Manzhynski S Biedenbach G. The knotted paradox of coopetition for sustainability: investigating the interplay between core paradox properties. Ind Mark Manag. (2023) 110:31–45. doi: 10.1016/j.indmarman.2023.02.013

[44] Raza-Ullah T Bengtsson M Kock S. The coopetition paradox and tension in coopetition at multiple levels. Ind Mark Manag. (2014) 43:189–98. doi: 10.1016/j.indmarman.2013.11.001

[45] Vătămănescu E-M Mitan A Andrei AG Ghigiu AM. Linking coopetition benefits and innovative performance within small and medium-sized enterprises networks: a strategic approach on knowledge sharing and direct collaboration. Kybernetes. (2022) 51:2193–214. doi: 10.1108/k-11-2020-0731

[46] Shiffman J. Agency, structure and the power of global health networks. Int J Health Policy Manag. (2018) 7:879–84. doi: 10.15171/ijhpm.2018.71, 30316239 PMC6186462

[47] Mclean JE Behringer BA. Establishing and evaluating equitable partnerships. J Community Engagem Scholarsh. (2022) 1:66–71. doi: 10.54656/nyqp1665

[48] Challenges, G. C. O. E. T. A. S. Evidence Commission Update 2023: Strengthening Domestic Evidence-Support Systems, Enhancing the Global Evidence Architecture, and Putting Evidence at the Centre of Everyday Life. Hamilton: McMaster Health Forum (2023).

