# Supplementary material for: Breaking silos, building bridges: leveraging Global Collaborative Evidence Networks for global health impact
Source: Front Public Health. 2026 Jul 8;14:1837626. doi: 10.3389/fpubh.2026.1837626 (PMC13390631; doi:10.3389/fpubh.2026.1837626)
Supplement: Supplementary file 1 [file Data_Sheet_1.PDF]

Attachment 1: Driving the EBHC Agenda Forward: Strengthening Local and Global Evidence Ecosystems

Principal Investigator: Bianca Pilla

What is the project/program about, and who is undertaking the research?

Background

Evidence-based healthcare (EBHC) is a worldwide movement with hundreds of organisations and thousands of individuals working to ensure that healthcare practice, policy, and decision-making are informed by rigorous research evidence to improve health outcomes. The success of this global agenda, however, depends on individuals and organisations working together within functioning evidence ecosystems (Pilla et al. 2022).

Evidence ecosystems are the local, national or global communities of actors who commission, fund, produce, synthesise, disseminate, translate and use research evidence, the formal and informal relationships between them and the policies, practices and structures that facilitate and underpin their interactions (Pilla et al. 2023).

In 2023, we reached the midway point for the 2030 United Nations Sustainable Development Goals and a global commitment to ‘leave no one behind’. However, the global crises of humanitarian disasters, climate change and health pandemics are all impeding progress and exacerbating global health inequities, highlighting the urgency to strengthen local and global evidence ecosystems to address these societal challenges (Jordan & Pilla 2024).

The JBI Collaboration, Cochrane Collaboration, Campbell Collaboration and many other Global Collaborative Evidence Networks (GCENs) have a unique role to play in bringing together diverse actors from within evidence ecosystems to address global health agendas, facilitating information sharing and enabling growth in shared capacities, providing opportunities to exploit existing knowledge, gain access to novel ideas, provide access to training, expertise and a platform for understanding global health issues and practical knowledge of how to solve complex health problems (Pilla et al. 2022).

Aims

JBI is developing a research program on the role of Global Collaborative Evidence Networks in responding to global health agendas and strengthening local and global evidence ecosystems.

In the first instance, this research program seeks to understand how Global Collaborative Evidence Networks work together, identifying opportunities for strengthening ties between and/or collaborating across GCENs

Bianca Pilla (Director, Global Relations, JBI) and Prof Zoe Jordan (Executive Director, JBI) are leading this program.

Why am I being invited to participate, and how long will it take?

As a Director or Convenor of a JBI Collaborating Entity, you are being invited to complete an online survey that will take **approximately 15 minutes to complete.**

What are the potential benefits of the research project/program?

It is anticipated that this program of research will contribute to strengthening ties between collaborative evidence networks and reducing duplication of effort across networks, produce guidance for research prioritisation in line with global health agendas and support the use of evidence-based models and frameworks for more coherent, coordinated, and collaborative approach to the delivery of evidence-based healthcare for global impact within and across local and global evidence ecosystems.

Are there any associated risks, and can I withdraw my participation?

Participation in this project is completely voluntary. If you agree to participate, you can withdraw from the study up until the submission of the survey.

Non-participation or withdrawal from the study will not affect your ongoing relationship with JBI.

What happens with my information?

Confidentiality and privacy: Participation in this survey is completely anonymous, and we will not be collecting any identifying information other than the country and sector within which you work.

Storage: Data collected as part of this project will be securely stored on a University of Adelaide server for no more than 7 years and will only be accessed by the Co-investigators for analysis.

Publishing: Information will be collated and analysed as part of a larger research program. It will be presented at the JBI Collaboration 70th Meeting 2023 to inform panel sessions and, if accepted, submitted for publication as journal articles and conference proceedings.

Survey analyses may be shared with others in future for the conduct of comparative research projects, and thus, consent provided here will apply to future use cases also. However, as no personal data is being collected, your responses will not be identifiable.

Who do I contact if I have questions about the project?

Bianca Pilla	**Ph:** +61 8 83133637	**Email:** Bianca.pilla@adelaide.edu.au
Prof Zoe Jordan	**Ph:** +61 8 83132926	**Email:** Zoe.jordan@adelaide.edu.au

What if I have a complaint or concerns?

This research project will be conducted according to the NHMRC National Statement on Ethical Conduct in Human Research 2007 (Updated 2018). If you have questions or problems associated with the practical aspects of your participation in the project or wish to raise a concern or complaint about the project, then you should consult the Principal Investigator. If you wish to speak with an independent person regarding concerns or a complaint, the University's policy on research involving human participants, or your rights as a participant, please contact the Human Research Ethics Committee's Secretariat on:

Phone: +61 8 8313 6028 Email: hrec@adelaide.edu.au **Post**: Level 3, Rundle Mall Plaza, 50 Rundle Mall, ADELAIDE SA 5000

Any complaint or concern will be treated in confidence and fully investigated. You will be informed of the outcome.

If I want to participate, what do I do?

By completing the survey, you are indicating that you are at least 18 years old, have read this consent form and agree to participate in this research study. Please print a copy of this page for your records.

Yours sincerely,

Bianca Pilla

Prof Zoe Jordan
