# Supplementary material for: Breaking silos, building bridges: leveraging Global Collaborative Evidence Networks for global health impact
Source: Front Public Health. 2026 Jul 8;14:1837626. doi: 10.3389/fpubh.2026.1837626 (PMC13390631; doi:10.3389/fpubh.2026.1837626)
Supplement: Supplementary file 2 [file Table_1.docx]

Supplementary Material 1

**JBIC Member Survey**

**Survey Questions**

*Denotes required answer

1. *In which country are you located? [Free Text Field]
2. *What sector do you work in?

a. Clinical (hospital/community)

b. Academic (university/college)

c. Government/Policy

d. Professional Society/Association

e. Other (please specify)

1. *Are you a member of, or affiliated with, other Global Collaborative Evidence Networks (GCENs)? Please select all that apply
2. Cochrane Collaboration
3. Campbell Collaboration
4. Guidelines International Network
5. Africa Evidence Network
6. World Health Organisation Collaborating Centre
7. World Health Organisation EvipNET
8. Other [Please Specify]
9. No

[Survey logic if selected A-G]

3.1* Do you feel that these GCENs foster a culture of open communication and knowledge sharing across networks? [Yes/No/Unsure]

3.1.1 Please elaborate on your response [Free Text Field]

3.2*What barriers or challenges do you encounter when collaborating with these GCENs? Please describe [Free Text field]

3.3* What specific characteristics, strengths, or initiatives from your interactions with these GCENs would be useful for JBI to consider? Please describe [Free Text field]

3.4* Based on your experience working with these different networks, do you see any opportunities for closer collaboration with JBI? [Free Text field]

[Survey logic if selected H]

1. *Do you feel that JBI fosters a culture of open communication and knowledge sharing across GCENs? [Yes/No/Unsure]
   1. Please elaborate on your response [Free Text Field]
2. *Do you feel that there are sufficient opportunities for collaboration across GCENs? [Yes/No/Unsure]

5.1 Please elaborate on your response [Free Text Field]

1. *Do you feel that there is duplication of effort across GCENs? [Yes/No/Unsure]
   1. Please elaborate on your response [Free Text Field]
2. *What roles can individual members of GCENs play in building bridges across networks? Please describe [Free Text field]
3. *What roles can leadership teams of GCENs play in building bridges across networks? Please describe [Free Text field]
4. *What initiatives or changes do you suggest that JBI could make to enhance collaboration with other GCENs? Please describe [Free Text field]

Are there any other comments that you would like to add regarding your experience in collaborating with JBI, other GCENs or working across networks [Free Text field]
