# Supplementary material for: Breaking silos, building bridges: leveraging Global Collaborative Evidence Networks for global health impact
Source: Front Public Health. 2026 Jul 8;14:1837626. doi: 10.3389/fpubh.2026.1837626 (PMC13390631; doi:10.3389/fpubh.2026.1837626)
Supplement: Supplementary file 3 [file Table_2.docx]

Supplementary Material 2

**JBIC Director Panel Questions**

1. You have each been invited to participate in this panel as you wear multiple hats across different collaborative evidence networks, including co-hosting different groups. Could you each briefly describe the global collaborative evidence networks (GCEN) with which you participate?
2. What was the motivation to host and or join different networks?
3. Can you provide an example of something you are proud of that you have accomplished in this multi-collaboration context?
4. What challenges and/or barriers have you faced in this work?
5. Reflecting on some of the suggestions from JBIC colleagues around opportunities for GCENs to collaborate more closely, what are 1 or 2 immediate concrete steps that could be taken by JBI and/or by JBIC entities?
6. What are some of the gaps not currently being filled by any evidence network that JBI, Cochrane, Campbell, GIN, etc., should or could be doing together?
7. If you had a “magic wand” what would be your desired outcome from the interaction of GCENs working collaboratively?
