# Supplementary material for: Breaking silos, building bridges: leveraging Global Collaborative Evidence Networks for global health impact
Source: Front Public Health. 2026 Jul 8;14:1837626. doi: 10.3389/fpubh.2026.1837626 (PMC13390631; doi:10.3389/fpubh.2026.1837626)
Supplement: Supplementary file 5 [file Table_4.DOCX]

# Survey & Panel Combined Thematic Analysis

## Challenges

## Theme: Navigating organisational, methodological and resource barriers working across GCENs

This theme captures the challenges faced when working across multiple GCENs, including organisational, methodological, and resource barriers, as well as the difficulties in communication and collaboration. This theme highlights the need for more strategic collaboration, improved resource allocation, and system harmonisation across GCENs to address the inherent challenges.

| **Code** | **Examples** |
| --- | --- |
| Gaps are prevalent in each GCEN, requiring different collaborations | “Policy for example is a far objective within JBI. But you can get approaches for policy within like the Africa Evidence Network. So, this is how we found interest in moving here and there.”  “And it was the fact that there were identified gaps in where as a JBI centre alone, without an additional affiliation or an additional organisation perhaps, we either weren't able to fill the gaps or maybe weren't able to be at the table to be part of the conversation around the gap-filling. Whether that's in terms of resources, the US system is very different in the way a lot of healthcare is funded.” |
| GCENs each experience organisational challenges | “The challenge is, is interesting, because in the end, networks at some point, will go through some challenges, yeah. When we joined Cochrane, it was very rich organisation with solid, very solid funding and all. And suddenly then, they went through quite a few challenges and issues needed to overcome… and I remember we also went through some challenges here in JBI… Remembering as now the Singapore meeting.” |
| Working with and across multiple networks requires increased time and human and financial resources | “If you are on many networks, you now have to be able to spread yourself out to be at the different conferences, to also provide reports to the different networks. Because you don't just go there to grab, you also go there to give, it's a two-way thing. So, one of our biggest challenges was staffing. And I think it's a challenge that we are still not able to beat… And the staffing also goes with budgeting. You also need bigger budget if you have to travel to these conferences, especially if the conferences are not funding your trip out there. So, these are all considerations that we have and we are finding different ways to beat them, but it's not so easy to beat them at this point.”  “There's very little, if any, governmental funding for some of the work that we do in this regard in the US. And so, that was the second piece of the motivation was positioning our Centre to be able to procure external funding for sustainability.”  “The only challenge is time and time management.”  “There are no barriers that could be controlled by JBI. We could say that there are barriers that should be managed by us, such as limited time and language.” |
| Duplication of effort across GCENs | “Although the topics may be slightly different, the systematic review process is duplicated across GCENs, with some different requirements.”  “At the last GIN, I don't remember the topic, but at a certain moment, I thought, we do this already at JBI.”  “Each GCEN has their respective methodologies and methods which can be frustrating and harmonization or choosing to focus on a limited set of methodologies for each entity bears discussion”  “Everyone has software for data management, tools for appraisal, would be nice to have an agreed system.” |
| Different operational systems across different GCENs (i.e. KPIs, reporting) | “And for example, we have used a lot of resources within JBI, but being able to keep track or keep pace with what's expected in terms of reports has been quite challenging…. And this cuts across different networks.”  “Our biggest challenge are the various requirements for reporting to KPIs, outputs, et cetera, to each of the individual entities [networks]. And where something may count on the JBI matrix, it doesn't give us any credit or standing with Cochrane or what may give credit in standing with Cochrane is not recognised within a JBI matrix. And when you're in a time and money pressure, we all have limited time and limited money to do our work, then it does require, unfortunately, at least for me, and I haven't figured out how to do this better.  “Different reporting standards/requirements to maintain membership/center status.” |
| Access to resources within networks is restricted/prohibitive | “But I think one aspect that really needs to be pushed forward within the perspective of equity, diversity, and inclusion is a consideration on the fees to accessing the resources of networks. It's not like zero fees, but what it means when maybe Zoe can pay $57 for a paper and I have to also pay $57. It means that following a purchasing power parity, I am paying five times more than Zoe. So, these are some considerations. I know we are not talking to JBI on this, but to the publishers. But it's at least this is the forum where I can speak about that.”  “There is a lack of access to information and resources in different networks.” |
| Infrequent communication/passive approach to collaboration/ lack of awareness across GCENs | “[A challenge to working across GCENs] is infrequent communications and very passive approach to collaboration across networks”  “I think that currently the GCENs operate individually with little collaboration other than the global evidence summit, but it is changing. “  “No-one really talks to each other and so I'm sure there is much work going on that we don't know about.”  “Opportunities [to collaborate] are not explicitly visible in communicated activities by GCENs” |
| GCEN members and organisations are working in silos | “I just find everyone is still working in silos I think there is more collaboration but there is still more to do together.”  “I had really optimism when in South Africa [Global Evidence Summit] but I still have not seen the level of collaboration I thought I would see.”  “I perceive these organisations [GCENs] as separate islands.” |
| Methodological differences across networks making collaboration challenging | “I have expended my efforts in learning JBI methodology and methods and do not have the time to tailor this across GCEN's”  “Methodological differences, for example the Cochrane's qualitative review methodology is very different and more complex than that of JBI. This makes collaboration challenging.” |
| Politics at the network/strategic management level | “The strategic management level has become so political and goal oriented in terms of their priorization that intellectual debates with those working in the margins are carefully avoided and close to impossible. The politics have come to hinder much of the early intellectual work that slipped into the organisations. The general tendency is: if you're not part of the mainstream or if you have a dissonant voice, move aside.”  “There are political/historical issues with GCENs that we may feel unsure about.” |
| Evidence syntheses not reaching all end users/developed with end users in mind | “Unless and until we reach to that place where bedroom talks are translated into boardroom talks, so that they [patients] feel empowered, they feel that, oh, there is an organisation, there is someone credible who is developing evidence for us, where they can go and have a look and decide for themselves what is good and what is bad, what is cost-effective, what is clinically effective. So, as networks, we need to reach out to that level where people look forward to us.”  “I think that was because we found a gap in the systematic reviews that were developed. Most of them were targeting practitioners, researchers, and sometimes policymakers completely leaving out those who actually need research, who are the public or the populations or the citizens. So, that's the gap I believe we should look at across networks as well.” |

## Facilitators

## Theme: Fostering synergy, collaboration and knowledge sharing across GCENs

This theme captures the importance of creating a cohesive and cooperative environment where individuals and groups within GCENs act as ‘bridges’ to share information, leverage diverse perspectives and expertise to enhance collaboration and overcome challenges. This theme underscores the importance of mutual understanding, interconnected roles, shared goals, and collaborative efforts in maximising the potential and impact of GCENs.

| **Code** | **Example** |
| --- | --- |
| Members working across GCENs as bridges/brokers | “For us, it's been an incredible opportunity having been a JBI Centre for a number of years and being newer to the Cochrane collaboration to see the stark similarities and clear different differences between the two organisations. And I think Bianca said it earlier, we've really been trying to be almost a mediator… to bridge the gap of around resource and resource waste.”  “The members of the centres can make a major contribution to the development of collaboration between the different networks by promoting the philosophy and methodology of JBI (ambassador of evidence), identifying the best and most interesting evidence available (JBI, Campbell or Cochrane), implementing evidence based practice at the point of care (auditor, leader), identification of unsafe practices and their de-implementation (auditor, opinion leader, professional model, role in change management, negotiator), etc.”  “Members create individual level connections with other members. This can also help fostering organizational level collaboration.”  “Individual members can expand the scope of GCENs and understand the situation in different environments.”  “Members create individual level connections with other members. This can also help fostering organizational level collaboration.”  “Individuals can advocate for GCEN's to explore bridge building activities. An example could be co-designing a shared tool.”  “The individual members can share the knowledge learned in one GCEN with another to improve the partnership and the way of working as a collaborative group.” |
| Commitment to sharing information and maintaining open/ transparent communication | “Communication is the core of the processes through which collaboration takes place. Both forms of practiced communication are important: formal communication (team meetings, rounds, charts) and informal communication (corridor chats and email exchanges). It is necessary for communication to be based on protocols and collaboration agreements and to be regular, active, reciprocal and open because both parties must be comfortable communicating with each other- this is how we succeed in working across GCENs.”  “Yes, interaction enables sharing of experiences including challenges and best practices”  “There are sufficient opportunities to collaborate with GCENs as long as there is space for communication.” |
| Enhancing communication through face-to-face meetings | “Yes, because physical interaction through meetings will enable more sharing across networks because of increase in trust and commitments.”  “When people meet physically they tend to be more reassured and encouraged.” |
| Partner values, purpose and/or interests aligned | “Also, for a successful collaboration, the actors must have common goals and a common purpose. They should have congruent philosophies and values and a commitment to mutual collaboration and cooperation. The process of forming collective goals is possible through dynamic team communication and negotiation. Consensus like an implicit understanding of how team members should work together is also essential.” |
| Working across GCENs for the same aim, bringing different perspectives | “And in all these three roles [across GCENs], what we do, we promote evidence-based nursing and also midwifery from different perspectives. So, we are doing a lot from different perspectives, but I think this all gives synergy.” |
| Working across GCENs to leverage existing expertise and capacity | “Collaboration with JBI is fantastic! Collaboration with other networks is also fantastic. Thanks to this collaboration we are learning so much and now we are already able to contribute with gain knowledge in different GCENs.”  “We were looking at reports from observational studies and we thought that they were not very competitive with those that had used the CONSORT-Equity. So, we reached out to the different networks, and we don't have the capacity or the funding to invest in this kind of study… we were able to set up a working group that started work on STROBE-Equity. And we will use these resources once it is developed… something which otherwise would not have been possible without the networks.”  “We are focusing on each three roles to the same agenda is how to promote evidence-based nursing and evidence-based healthcare. So, I think that's one thing that helps us, because we can use the information from one side to help the other one. And so, that's maybe the key thing that helps us to move forward.” |
| Learning how to overcome challenges from different networks | “So, we say now these experiences are helping solutions, because if we see something work out somewhere else, it should work in other collaboration as well. So, that's how I see it. It's rather the benefit actually seeing how they [different networks] can overcome the challenges.” |
| Working on joint events and global initiatives drives collaboration across networks | “This [panel] session is example of it, I would say. So, more of these sessions I think would be important for the future of networks working together.”  “The first Global Evidence Summit, the JBI GIN conference, the second Global Evidence Summit, World Evidence-Based Healthcare Day. I think those are all very good examples. And also, what we've seen in the first session that actually is JBI was putting up the grant to see how these collaborative entities can actually collaboratively approach towards the SDGs. That's something what I see is going on here. So, I would just say, eh, let's continue.”  “Yes - the World EBHC international day encourages this [open communication and knowledge sharing across GCENs]”  “I have collaborated conference regularly, join other GCENs' methodology committees, and invite other GCENs join JBI's methodology committee.”  “Global Evidence Summit is a great example of collaboration between JBI, Cochrane, GIN and Campbell. There could be perhaps more activities which can build on the GES.” |
| Willingness to work together (co-design, co-build, co-contribute) | “The networks that I am involved are open for collaboration in the area of EIDM- there is a willingness to work together.”  “Yes, because there are collaborative projects that involves members from different institutions. For example, i am with JBI but have work on reviews with members from the Cochrane qualitative research group- I find people from different networks are happy to contribute.” |
| Understanding and valuing each Network's strengths, limitations, perspectives and contributions | “I think it is very important that JBI continue to focus on their point of difference from other GCEN's i.e., methodologies for different evidence syntheses, evidence implementation and point of care resources. Each GCEN has its own strengths.”  “Multiplication of meetings encourages members. Efforts are complementary rather than contradictory.”  “Because each GCENs has their own history, mission and vision and strengths.”  “Each networking has its own unique features.” |

## Benefits

## Theme: Having ties with multiple GCENs yields benefits

This theme emphasises how working across multiple GCENs enhances the overall reach, effectiveness, and resource availability for Evidence-Based Healthcare activities and highlights the synergistic benefits of collaboration across diverse networks, enhancing the ability to address global health challenges more effectively.

| **Code** | **Example** |
| --- | --- |
| Multiple GCENs amplify reach and impact of EBHC activities | “All these networking collaborations, they helped us to reach out to millions. If there wasn't a collaboration, I wouldn't have been standing here and talking to you and sharing my experience. So, that is a potential in multiple collaborations”  “We have the National Institute for Excellence and Quality in Healthcare, which is the institute built by the Ministry of Health… it wouldn't be possible without actually having a very strong international collaboration with different organisations behind us to help us to make these changes in our country”  “Having a collaboration with WHO, JBI and others, we were able to save lives. So, during COVID, there was a call from Somalia, they didn't have electricity in their healthcare Centres… we need to develop an oxygen concentrator with solar energy…. But that was only possible when we had a collaboration and we had different centres, dedicated centres for technology… and building an evidence around all these innovations, process modifications and everything. So, that is an immense potential. That is a model where we can show what was the result of this collaboration and what we are able to achieve now- and this was South-South collaboration”  “In addition we have richer experience, projects, publications and other output in EBHC because of participation in different networks, that made us feel confidence in international EBP family.” |
| Different resources/support from different networks | “What I would say is that various needs also need various resources. And that's why we find ourselves in so many collaborations.”  “And this is the philosophy that we've used within these networks, and it's been extremely useful. I would say the networks are like money in the bank if you know how to use them.”  “So, we found ourselves in a situation where we rolled out a JBI audit and feedback intervention in the treatment of malaria, and it increased the knowledge asymmetry between the clinicians and the policymakers. So, it meant that we needed something more than just audits and feedback…Policy for example is a far objective within JBI. But you can get approaches for policy within like the Africa Evidence Network. So, this is how we found interest in moving here and there.”  “And of course, but then, Nikez as I spoke about, it’s something very, actually, we used the fruits from all the different collaborations [networks] and combine it in one institute.” |
| Different networks connect individuals with diverse perspectives, knowledge, and skills | “We have similar reasons why we have been taking part in these networks [including] to gain the different ideas, information, what's happening within evidence-based healthcare and keeping us up-to-date, and then transferring this information also to our national role… lots of people know lots on different things. So, the collaboration is one key thing. So, once we know who to contact, what's happening in different countries, if we are for example, planning some international projects or something like that, that helps a lot when you know people from different countries and networks.”  “General knowledge sharing, working with other networks learns me to put my knowledge in perspective. I understand where, for me is the place for JBI and where do I prefer other methodologies.” |
| Collaboration with multiple networks aids with recognition and funding | “Taking part in these different networks, we think that the politicians also see us more valuable for the national purpose as well. And for example, when we are talking with the politicians and trying to explain why we need these different resources.”  “There's very little, if any, governmental funding for some of the work that we do in this regard in the US. And so, that was the second piece of the motivation was positioning our centre to be able to procure external funding for sustainability. Because let's face it, at the end of the day, this work is amazing and wonderful, and a lot of us do this work in kind and for free, but that's not a sustainable model for the long-term.” |
| Learning from different challenges, structures, systems and operational frameworks | “when we started the evidence-based research network, the colleagues, because they knew we are collaborating with a lot of entities asking, "could you please do the analysis for us to know what are the business models and what are the challenges, opportunities, how they all work?" And it was quite interesting for myself actually to go through all the structures and relate this. So, yeah, really, I still see the synergy between the different networks.” |
| Increase shared meetings, and collaboration on events and global initiatives | “JBI should open up its conferences and symposiums to other evidence synthesis institutions”  “Yes I think for future conferences the organizations should get together and present a more unified front for example meet the first day plan sessions together work more together to advance science in this area for all.”  “I think conferences are a great place I am just not sure this has been done to the level it could be.”  “Having regular meetings in planning and implementing collaboration [across GCENs].”  “Continue to host summits or conferences where the GCEN's combine and promote a shared cause/vision. Open discussions about different GCEN's and how they can contribute to the global community.”  “Collaborative meetings with representatives from various groups to present to the same audience.”  “Collaborative meetings with multiple GCEN's. More online workshop opportunities.” |
| Cross-network collaboration for global health challenges | “The COVID-19 took us quite a lot of things, but also gave us something. And I was myself, involved in several amazing projects. One of the most impactful from my perspective was the COVID-19 map of recommendations. There were entities from around the around the globe, all with which we represent, but also many, many other, which I didn't know before working collaboratively, actually getting all existing guidelines for COVID-19 under one roof, under one platform.,, [it] was great to see how quickly you're able to actually work together without money in the first place, and actually then making sustainable, thanks to the grants. But yeah, so it'll be perhaps this, some umbrella where we should be able to identify where are our strengths and weaknesses to work collaboratively strongly together.”  “What I see in this growing collaboration between the collaborative evidence networks is the ability for us to meet that goal of evidence-based healthcare really in a broader way and not just meet the specific outcomes for each organisation, but truly create the global impact that we are all after.”  “Yes, participation in multiple networks provide us knowledge on topical global challenges and possibilities for international collaboration.”  “In questions and healthcare challenges that are global, it is useful to have possibility to consider joint statements from the JBIC /and other networks (directed to politicians) together with different participants.” |

## Opportunities

## Theme: Opportunities for harmonisation across GCENs: leveraging existing strengths, finding synergies

This theme encompasses the opportunities presented by working across multiple GCENs to enhance synergy, share resources, co-produce methodologies, and align systems for improved collaboration. This theme underscores the potential for creating a more integrated, structured and efficient collaborative environment to harness the collective strengths of various GCENs for greater impact.

| **Code** | **Example** |
| --- | --- |
| Network synergy- combining strengths and benefits from multiple networks | “It's really different collaborations, different opportunities, and we find a way how they actually are in a synthesis altogether. And for me, one of the very important topics and one of the important themes we have on a global evidence summit is the power of synergy.”  “And that's exactly what actually we were seeking, and for our main goal to actually change the national policy, to establish the National Guideline Institute, it was really that we thought, well, okay, we need a lot of the support to change something in a very stubborn country like it's our country. You need really this strong advocation from all sides.”  “I think we don't need more international organisations. We need the one which will be effectively connecting all we have. Yeah, because sometime, it's happening and I see it now, again, mentioning Global Evidence Summit. Learning a lot from this experience truly, because there are many great networks who are, and we even don't know they exist here until we find them. And I think really that what we perhaps need in this is really some harmonisation and able to find where are the strengths of each of these collaborations and combine them, or have some umbrella organisation, which would work this way”  “so it'll be perhaps this, some umbrella where we should be able to identify where are our strengths and weaknesses to work collaboratively strongly together.”  “I might be a bit idealistic, but there are many global problems within the healthcare. There is the shortage of healthcare staff, mental health issues, retention, and so forth. So, maybe this kind of umbrella could help us put the resources together to find evidence-based answers to these challenges and make our politicians in different countries to understand that what is the best solution to overcome these challenges. So, maybe that kind of umbrella would be something that would help us working together to solve these big problems.”  “As they [GCENs} express their strengths and challenges, there is need to complement each other as well as learning from others challenges and complying good practices.” |
| Opportunity to work across GCENs on EBHC methodology | “And I'm really, really proud of the fact that so much of the JBI methodology and model has been infused into that work where otherwise it would not have been at all. So, I think it's really been a tremendous opportunity to share between JBI, Cochrane, and Campbell in particular in this work around this methodology.”  “I think there's a lot of opportunity around methods and methodology… JBI has some fantastic methodology, but doesn't necessarily have to be the leading expert on every single methodology possible. Rather, take the lead on those that are really within our wheelhouse at JBI and collaborate with others where it's really within their wheelhouse. Be it Campbell or Cochrane or GIN or fill in the blank of which opportunity. I think the inclusive nature of JBI from the onset really positions JBI to be the leader of helping those collaborations and community of methodological science development work together… I think there's a room there rather than duplication of methodological development and support in silos across. And I know that's already being done some, but I think there's a lot of opportunity to grow that.”  “Method development across GCENs should be consolidated. Duplication of efforts.”  “GCEN entities may consider either harmonizing methodologies and methods or each GCEN select a set of methodologies and methods as their focus (reduce duplication of effort).”  “Work across groups for method development, topic development, dissemination and implementation.” |
| Considered effort for joint individual and network level international/multidisciplinary projects | “Yes, perhaps share review capacity across collaborations as the topical areas we engage with are pretty much similar. I dream of one big, open access database where all protocols and systematic reviews can be found, rather than each organisation having their own. Wouldn't it be nice if we could just sign up for a project at intention letter level to establish diversity in perspectives across review author groups?”  “Increase collaboration, Work on joint projects, Members should participate in activities of other institutions if given the opportunity.”  “Creating opportunities for joint projects and ways of working together.”  “Involvement in international, multidisciplinary projects.”  “The sky is the limit. They could all be conceptualizing small to large scale collaborative activities. Try writing a grant together.”  “Participation in joint projects such as new methodologies developing (participation in working groups), developing existing methodologies, systematic analyses developing, the promotion/diffusion of working together in different events, organizing and running meetings, events, and workshops with special guests from other GCENs, the identification of people with skills in systematic analyses, the training of people with skills and availability to work in the field of systematic analyzes and evidence implementation…” |
| Create formal pathways for sharing information and resources between GCENs | “imagine if we have a way, a pathway where model evidence is being generated in JBI, directly goes to WHO or directly goes to different federal governments where they can make it applicable to on ground, see the immense potential in that.”  “imagine a potential where if we have a page in JBI website where we all GCENtres put our information, what is happening across the globe, we need not to reach out to a hundred centres. We can reach out to JBI website and everything is there, which is catering to everybody's need. If we are doing any event, we publish that there. So, that's how I see a magical collaboration and that's how we were able to make a difference.”  “Learn and share with each other, open dialogue within networks.”  “Invite the other partners for your events - communicate open about your plans and activities.”  “Coming together and reduce duplication of works or systems. Sharing of resources across GCENs”  “Advocating for resources and best practice sharing.” |
| Create pathways, systems and agreements for collaboration across networks | “Create formal agreements and pathways for collaboration.”  “Give enough leadership support or build some policies to support working across networks.”  “Restructure to invite wider membership from across GCENs.”  “This is where I see a greater opportunity to leverage our collective resources and create opportunities for sharing through possibly joint memberships or collaborating groups.” |
| Promoting opportunities for collaboration across GCENs | “Promoting GCEN's across own networks.”  “identify areas of expertise needed/lacking in projects and publicize across GCENs”  “Promote awareness of opportunities”  “Facilitating open communication for opportunities. Seeking partners for international grants. Sharing expertise.” |
| Better align membership terms/requirements/ systems across GCENs | “we're coming up on maybe looking at the JBI matrix over the next few years. And so, to the extent that we collectively can think about how we collaborate well with recognition and appropriate points or whatever metric we're using to give that work value as it relates to important outputs that need to occur from Cochrane, GIN, Campbell, JBI, et cetera” |
| Platforms for registering and sharing data, projects, software etc. | “Common data management software. Table generating software that meets the expectations of the editors. So much editing by authors after these tables are generated.”  “Registrations of SR protocols. Some topics of SR may be duplicated. Some implementation and advocation activities are duplicated.” |
| Role of GCENs in co-production/ knowledge user engagement | “I don't know that any of the various entities [networks that] engage with the end users as well as we should…But what we don't do, in my assessment, across any of the networks, is a good enough job engaging those that are ultimately impacted by the work that we produce. And I think there's a tremendous opportunity with the expertise of everybody in this room and elsewhere to figure that out. It's not an easy solution, I don't think, but so incredibly important.”  “We are not reaching to patients what exactly they want. So, what are we doing? Where we are not involving them for policy decisions. That's how we turn the table and that's how we now modified the way of working. We reach out to them, we take a feedback from them, and then we develop something for them. So, it's very important. And as far as how we can contribute and how we should do that is matchmaking… there's a way to reach out to different networks where we have already established networks... The only thing is we need is to connect.” |
